# Tracking Parkinson’s Disease over One Year with Multimodal Magnetic Resonance Imaging in a Group of Older Patients with Moderate Disease

**DOI:** 10.1371/journal.pone.0143923

**Published:** 2015-12-29

**Authors:** Tracy R. Melzer, Daniel J. Myall, Michael R. MacAskill, Toni L. Pitcher, Leslie Livingston, Richard Watts, Ross J. Keenan, John C. Dalrymple-Alford, Tim J. Anderson

**Affiliations:** 1 New Zealand Brain Research Institute, Christchurch, New Zealand; 2 Department of Medicine, University of Otago, Christchurch, New Zealand; 3 College of Medicine, University of Vermont, Burlington, VT, United States of America; 4 Christchurch Radiology Group, Christchurch, New Zealand; 5 Department of Psychology, University of Canterbury, New Zealand; 6 Department of Neurology, Christchurch Hospital, Christchurch, New Zealand; University of Toronto, CANADA

## Abstract

**Background & Objectives:**

Cross-sectional magnetic resonance imaging (MRI) suggests that Parkinson’s disease (PD) is associated with changes in cerebral tissue volume, diffusion tensor imaging metrics, and perfusion values. Here, we performed a longitudinal multimodal MRI study—including structural, diffusion tensor imaging (DTI), and perfusion MRI—to investigate progressive brain changes over one year in a group of older PD patients at a moderate stage of disease.

**Methods:**

Twenty-three non-demented PD (mean age (SD) = 69.5 (6.4) years, disease duration (SD) = 5.6 (4.3) years) and 23 matched control participants (mean age: 70.6 (6.8)) completed extensive neuropsychological and clinical assessment, and multimodal 3T MRI scanning at baseline and one year later. We used a voxel-based approach to assess change over time and group-by-time interactions for cerebral structural and perfusion metrics.

**Results:**

Compared to controls, in PD participants there was localized grey matter atrophy over time in bilateral inferior and right middle temporal, and left orbito-frontal cortices. Using a voxel-based approach that focused on the centers of principal white matter tracts, the PD and control cohorts exhibited similar levels of change in DTI metrics. There was no significant change in perfusion, cognitive, or motor severity measures.

**Conclusions:**

In a cohort of older, non-demented PD participants, macrostructural MRI detected atrophy in the PD group compared with the control group in temporal and orbito-frontal cortices. Changes in diffusion MRI along principal white matter tracts over one year were found, but this was not differentially affected by PD.

## Introduction

Detailed information on progressive brain changes is needed to help assess novel Parkinson’s disease (PD) modifying therapies. Progression based solely on clinical assessments is valuable, but unlike objective biomarkers, clinical measures cannot differentiate between symptomatic relief and neuroprotection. Structural MRI has provided biomarkers in neurodegenerative diseases such as Alzheimer’s disease (AD) [1], while cross sectional MRI studies in PD have identified robust brain differences associated with both clinical and cognitive measures [2–8]. Serial imaging predicts progression in progressive neurodegenerative diseases [9–11], and therefore may also track progression in PD.

Studies have reported progressive brain atrophy in PD over 1–4 years, but it is uncertain whether atrophy is significantly greater in PD than that seen in healthy ageing subjects [12–23]. Moreover, the value of serial diffusion [24] and perfusion MRI is relatively unexplored in the context of PD progression. Here, we performed a one year longitudinal multimodal MR imaging study—including structural, diffusion, and perfusion MRI—to investigate progressive brain changes over one year in a group of 23 non-demented, older, PD patients at a moderate stage of disease and 23 healthy control volunteers. We chose the relatively short one-year interval for reasons of applicability in research and clinical practice. Large-scale clinical trials examining potential therapies for neuroprotection are expensive and the establishment of an objective marker that can show clinically meaningful disease progression—and lack of progression due to an effective therapy—over just one year, would therefore be highly relevant for intervention studies.

## Methods

### Subjects

A convenience sample of 27 participants meeting the UK Parkinson’s Disease Society’s criteria for idiopathic PD [25], were recruited from volunteers at the Movement Disorders Clinic at the New Zealand Brain Research Institute, Christchurch, New Zealand. Exclusion criteria at baseline included atypical parkinsonian disorder; history of other neurological conditions such as moderate-severe head injury, stroke, dementia; and major psychiatric or medical illness (previous six months). Twenty-three healthy controls, free from a history of major neurological or psychiatric disorders were also recruited, matched to the PD group by mean age and education years, and sex ratio.

From December 2010 –August 2013, eligible subjects were invited to undertake neuropsychological testing, clinical assessment, and MRI scanning at baseline (Y0) and one year (± 1 month) later (Y1). All MRI scans at Y0 and Y1 were screened by a consultant neuroradiologist (RJK) to exclude significant non-PD cerebral pathology. At Y1, one PD patient was unable to participate due to a non-PD illness, two patients declined to participate at follow up, and one patient was excluded due to new left occipitotemporal and right thalamic infarcts. After visual inspection, excess motion necessitated removal of T1-weighted images from two PD patients assessed as showing normal cognition (PDN) at both timepoints and the perfusion scan from one patient who was PDN at Y0 but converted to dementia at Y1). No diffusion datasets were excluded. Hence there were 23 healthy controls for all imaging analyses, but a different number of PD participants across imaging modalities: 21 for grey matter, 23 for diffusion, and 20 for perfusion MRI (see Table 1 for characteristics of the final sample). All subjects gave written consent, with additional consent from a significant other when appropriate. The study was approved by the regional Ethics Committee of the New Zealand Ministry of Health (No. URB/09/08/037).

**Table 1:** Demographic and clinical data in PD and control groups at baseline.

	Control	PD
n	23	23
Age	70.6 (6.8)	69.5 (6.4)
Male:female	16:7	17:6
Education (years)	13.5 (2.3)	13.0 (3.0)
N:MCI:dementia Y0	21:2:0	19:4:0
N:MCI:dementia Y1	23:0:0	17:5:1
Disease duration from diagnosis (years)	-	5.6 (4.3)
Time between scans (years)	1.0 (0.07)	0.98 (0.12)

Data presented as ratios or mean (SD). PD = Parkinson’s disease; Y0 = baseline; Y1 = follow up; N = cognition in the normal range; MCI = mild cognitive impairment.

### Diagnostic Criteria and Assessment (Y0 and Y1)

Motor function was assessed using the Movement Disorders Society Unified Parkinson’s Disease Rating Scale (MDS-UPDRS: part 3) [26] and cognition by comprehensive neuropsychological testing [4, 27, 28]. PD patients were classified as either cognitively normal (PDN, n = 20), with mild cognitive impairment (PD-MCI, n = 4, MDS Task force level II criteria [29]), or dementia [30]. MCI cases had unimpaired functional activities of daily living, as verified by interview with a significant other, but scored 1.5 SD or more below normative data on at least two measures within at least one of five MDS cognitive domains (executive function; attention, working memory and processing speed; learning and memory; visuospatial/visuoperceptual function; and language; see S1 Table for a list of the tests used). Cognitive domain scores were derived from averaged standardized scores from constituent domain tests; global cognition was expressed by averaging four domain z-scores (language excluded due to low variance) to form a single mean cognitive z-score. At Y0, two control participants met criteria for MCI, however at Y1 both were classified as having normal cognition.

At Y0, four PD participants were drug-naïve with respect to anti-parkinsonian medication; three remained drug-naïve at Y1. In all other PD individuals, motor, cognitive, and MRI assessments were performed on medication, with no change to their usual drug regimen. Daily dopaminergic medications were standardized into a levodopa equivalent dose (LED) [31].

### Magnetic Resonance Imaging acquisition (Y0 and Y1)

Imaging was conducted on a 3T General Electric HDxt scanner (GE Healthcare, Waukesha, USA) with an eight-channel head coil.

#### Conventional structural

Volumetric T1-weighted (inversion-prepared spoiled gradient recalled echo (SPGR), TE/TR = 2.8/6.6 ms, TI = 400 ms, flip angle = 15 deg, acquisition matrix = 256×256×170, FOV = 250 mm, slice thickness = 1 mm) and clinical T2 and T2 FLAIR images were collected.

#### Diffusion tensor imaging

A 2D diffusion-weighted, spin echo, echo planar imaging sequence was used to measure microstructural integrity, with diffusion weighting in 28 uniformly distributed directions (*b* = 1000 s/mm^2^) and 4 acquisitions without diffusion weighting (*b* = 0 s/mm^2^): TE/TR = 86.4/13000 ms, flip angle = 90 deg, acquisition matrix = 128×128×48, reconstruction matrix = 256×256×48, FOV = 240 mm, slice thickness = 3 mm, reconstructed voxel size = 1.07×1.07×3 mm^3^, NEX = 1, ungated.

#### Arterial spin labelling

A stack of spiral, fast spin echo acquired images were prepared with pseudo-continuous arterial spin labelling and background suppression to measure whole brain perfusion quantitatively [32]: TR = 6 s, echo spacing = 9.2 ms, post-labelling delay = 1.525 s, labelling duration = 1.5 s, eight interleaved spiral arms with 512 samples at 62.5 kHz bandwidth and 30 phase encoded 5mm thick slices, NEX = 5, units: ml/100 g/min). Participants were asked to close their eyes.

### Structural MRI Preprocessing

For each participant, Y0 and Y1 structural (SPGR) images were aligned to a subject-specific midpoint-space between the two scans using the longitudinal registration utility in SPM12b (v5581, http://www.fil.ion.ucl.ac.uk/spm/), running in Matlab R2010a, using default values [33]. The mid-point average image for each individual was then segmented and grey matter (GM) atrophy rate images produced by multiplying the native space GM segments (both Y0 and Y1) by the Jacobian rate. We then ran DARTEL (existing template) using the template provided with the VBM8 toolbox (in MNI space, http://dbm.neuro.uni-jena.de/vbm/). GM atrophy rate images, as well as the mid-space average GM segments, were then normalized using the DARTEL flow fields, modulated, and smoothed (8 mm FWHM). A GM mask was created by averaging all normalized, modulated GM segments, thresholding at 0.2 and smoothing (8 mm). In addition, default parameters were used to estimate percent brain volume change over one year using SIENA (FSL v5.0.2, http://fsl.fmrib.ox.ac.uk/fsl/fslwiki/, note: there was no correction for intracranial volume as SIENA calculates percent volume change relative to baseline) [34].

### DTI preprocessing

Preprocessing and analyses were performed in FSL per Engvig et al. [35]. At each timepoint, this included motion- and eddy current distortion-correction; rotation of the b matrix accordingly; motion quantification via root mean square deviation between each pair of realigned diffusion images and averaging over all pairs to create a single, ‘relative’ motion metric; brain extraction; and fitting a diffusion tensor to produce fractional anisotropy (FA), mean diffusivity (MD), axial diffusivity (L1, the principal diffusion eigenvalue), and radial diffusivity (RD, the mean of the second and third eigenvalues) images. FA images for Y0 and Y1 were linearly registered (FLIRT), resampled into the space halfway between the two, and averaged to create a midpoint-space subject average FA. This was then entered into a tract-based spatial statistics (TBSS) analysis to create the group-wise FA skeleton (thinned at FA>0.27), representing the centers of all tracts common to all participants [36]. Midpoint-space registered FA images (Y0 and Y1) were smoothed (sigma = 2) to reduce residual misalignment between Y0 and Y1 images, and projected onto the skeleton to create separate FA skeletons at Y0 and Y1. Using the transforms derived from the FA procedure, we created separate MD, L1, and RD skeletons at each timepoint. Lastly, we created average and difference FA, MD, L1, and RD skeletonized images for each individual.

### ASL preprocessing

At each timepoint, quantified cerebral blood perfusion images were co-registered to the structural image. Structural images were brain extracted using BET (in FSL), and the resulting brain mask was used to exclude non-brain tissue in the ASL images. Deformation fields mapping either Y0 or Y1 to the mid-point average were combined with the DARTEL flow fields to normalize the quantified perfusion image at each timepoint. We then created average perfusion and difference images for each individual, which were smoothed (10 mm).

### Statistical Analyses

Clinical, cognitive, and global MRI measures were compared across time and group using linear mixed-effects models with the *nlme* package in R (v3.0.0). Global MRI metrics included (1) percent brain volume change (SIENA), and at each timepoint, (2) DTI metrics averaged across the white matter skeleton, and (3) average grey matter perfusion extracted from the GM mask. Baseline age, sex, education and time between scans were included in all models. Relative motion was included in the DTI models only.

### Voxel-wise statistical analysis

All voxel-wise comparisons were performed using a permutation-based inference tool for non-parametric thresholding (FSL’s “randomise”) [37]. Group, time, and group-by-time interactions were investigated for each of 6 imaging measures (GM volume, FA, MD, L1, RD, and perfusion). The group effect (controls vs PD) was tested by investigating an average image for each individual (two sample t-test); difference images were used to investigate time effects in each group separately (one sample t-test) and group-by-time interactions (via two sample t-test of difference images). For all comparisons, age at Y0, sex, and years of education were included as covariates. For perfusion and DTI comparisons, time between scans was entered as a covariate (this was incorporated into the preprocessing of GM volume); de-meaned baseline scans were included as voxel-wise covariates to account for potential baseline differences. Relative motion was entered as an additional covariate in the DTI models (average relative motion for the group comparison and difference in relative motion for time and group-by-time comparisons). For each contrast, the null distribution was generated over 5000 permutations and the alpha level set at p<0.05, corrected for multiple comparisons (family-wise error correction using threshold-free cluster-enhancement (TFCE)) [38].

## Results

By Y1, two PDN individuals met a worse cognitive criterion, one to PD-MCI and one to PDD. The two controls who met criteria for MCI at baseline reverted to the cognitively normal category by Y1.

Table 2 summarizes clinical and cognitive data at Y0 and Y1, while Table 3 presents global MRI metrics at both timepoints. At baseline, we identified cognitive impairment in PD relative to controls (global cognitive z-scores and all individual domain scores except Language were significantly lower, although MoCA scores were not). Relative motion during the DTI scan was not significantly different between the groups. Furthermore, there were no significant effects of time or group-by-time interactions upon cognitive z-score, the five cognitive domain scores, MoCA, or relative motion, and no significant change in UPDRS III or LED (Tables 2 and 3).

**Table 2:** Cognitive and clinical metrics at baseline and follow up.

	Control group		PD group		PD vs. Controls	
	Baseline	Change after 12 months	Baseline	Change after 12 months	Baseline difference	Relative change after 12 months
	Mean [95%CI]	Mean [95%CI]	Mean [95%CI]	Mean [95%CI]	Mean [95%CI]	Mean [95%CI]
Global cognition (z-score)	0.68 [0.45–0.90]	0.12 [-0.01–0.25], *p = 0*.*07*	0.22 [0.005–0.44]	0.04 [-0.09–0.17], *p =* 0.5	-0.45 [-0.74 –-0.16], *p = 0*.*003*	-0.08 [-0.26–0.10], *p = 0*.*4*
MoCA (point)	26.7 [25.7–27.7]	0.7 [-0.2–1.6], *p = 0*.*1*	26.4 [25.4–27.4]	-0.2 [-1.1–0.8], *p = 0*.*7*	-0.3 [-1.6–1.0], *p = 0*.*7*	-0.9 [-2.2–0.5], *p = 0*.*2*
**Cognitive Domain Scores**						
Attention	0.28 [0.02–0.54]	0.09 [-0.07–0.25], *p = 0*.*2*	-0.06 [-0.32–0.19]	-0.02 [-0.18–0.14], *p = 0*.*8*	-0.35 [-0.69 –-0.01], *p = 0*.*045*	-0.11 [-0.33–0.11], *p = 0*.*3*
Executive function	0.74 [0.43–1.06]	0.04 [-0.12–0.20], *p = 0*.*6*	0.23 [-0.07–0.54]	-0.05 [-0.21–0.11], *p = 0*.*5*	-0.51 [-0.91 –-0.10], *p = 0*.*02*	-0.09 [-0.31–0.14], *p = 0*.*4*
Visuospatial/perceptual	0.59 [0.33–0.84]	0.002 [-0.23–0.23], *p = 0*.*9*	0.19 [-0.05–0.44], *p = 0*.*1*	0.01 [-0.22–0.24], *p = 0*.*9*	-0.39 [-0.72 –-0.06], *p = 0*.*02*	0.01 [-0.31–0.34], *p = 0*.*9*
Learning & memory	1.1 [0.75–1.47]	0.34 [0.05–0.63], *p = 0*.*02*	0.55 [0.20–0.90]	0.15 [-0.15–0.44], *p = 0*.*3*	-0.56 [-1.02 –-0.10], *p = 0*.*02*	-0.20 [-0.62–0.22], *p = 0*.*3*
Language	0.24 [0.06–0.41]	0.01 [-0.15–0.18], *p = 0*.*9*	0.11 [-0.06–0.28]	0.03 [-0.14–0.19], *p = 0*.*8*	-0.13 [-0.36–0.97], *p = 0*.*2*	0.01 [-0.22–0.24], *p = 0*.*9*
**Within the PD group**						
UPDRS motor score (point)	-	-	32.6 [26.8–38.4]	-1.9 [-6.1–2.2], *p = 0*.*3*	-	-
LED (mg/day)	-	-	609 [410–809]	69 [–15–152], *p = 0*.*1*	-	-

MoCA = Montreal Cognitive Assessment, PD = Parkinson’s disease, UPDRS = Unified Parkinson’s Disease Rating Scale part 3.

**Table 3:** Global MRI metrics at baseline and follow up.

	Control group		PD group		PD vs. Controls	
	Baseline	Change after 12 months	Baseline	Change after 12 months	Baseline difference	Relative change after 12 months
Global MRI Measures	Mean [95%CI]	Mean [95%CI]	Mean [95%CI]	Mean [95%CI]	Mean [95%CI]	Mean [95%CI]
% brain volume loss / year (SIENA)	-	-0.48 [-0.70 –-0.25], *p<0*.*001*	-	-0.71 [-0.93 –-0.49], *p = 0<0*.*001*	-	-0.23 [-0.52–0.05], *p = 0*.*1*
GM perfusion (ml/100g/min)	42.3 [38.9–45.8]	0.2 [-3.1–3.5], *p = 0*.*9*	39.6 [36.0–43.2]	-0.8 [-4.3–2.7], *p = 0*.*7*	-2.7 [-7.3–1.9], *p = 0*.*2*	-1.0 [-5.8–3.8], *p = 0*.*7*
**DTI metrics from WM skeleton**						
FA	0.41 [0.39–0.42]	-0.002 [-0.004–0.0005], *p = 0*.*1*	0.41 [0.39–0.43]	-0.0003 [-0.003–0.002], *p = 0*.*8*	0.0007 [-0.01–0.01], *p = 0*.*9*	0.002 [-0.002–0.006], *p = 0*.*3*
MD (×10^−3^ mm^2^/s)	0.87 [0.83–0.91]	0.0026 [-0.0041–0.0093], *p = 0*.*4*	0.88 [0.85–0.92]	0.00032 [-0.0066–0.0073], *p = 0*.*9*	0.012 [-0.0085–0.032], *p = 0*.*2*	-0.0023 [-0.012–0.0073], *p = 0*.*6*
L1 (×10^−3^ mm^2^/s)	1.2 [1.2–1.3]	0.00064 [-0.0079–0.0092], *p = 0*.*9*	1.3 [1.2–1.3]	-0.00062 [-0.0095–0.0082], *p = 0*.*8*	0.017 [-0.0018–0.036], *p = 0*.*08*	-0.0012 [-0.013–0.011], *p = 0*.*8*
RD (×10^−3^ mm^2^/s)	0.68 [0.64–0.71]	0.0036 [-0.0026–0.0097], *p = 0*.*3*	0.68 [0.65–0.72]	0.00073 [-0.0057–0.0071], *p = 0*.*8*	0.0093 [-0.013–0.032], *p = 0*.*4*	-0.0028 [-0.011–0.0060], *p = 0*.*5*
Relative motion (mm)	0.46 [0.43–0.49]	0.0073 [-0.018–0.032], *p = 0*.*6*	0.47 [0.45–0.50]	0.019 [-0.018–0.023], *p = 0*.*5*	0.015 [-0.023–0.053], *p = 0*.*4*	0.012 [-0.023–0.048], *p = 0*.*5*

GM = Grey matter, DTI = Diffusion Tensor Imaging, FA = Fractional Anisotropy, MD = Mean Diffusivity, L1 = axial diffusivity, RD = radial diffusivity, PD = Parkinson’s disease.

### Global MRI metrics

**Fig 1:**
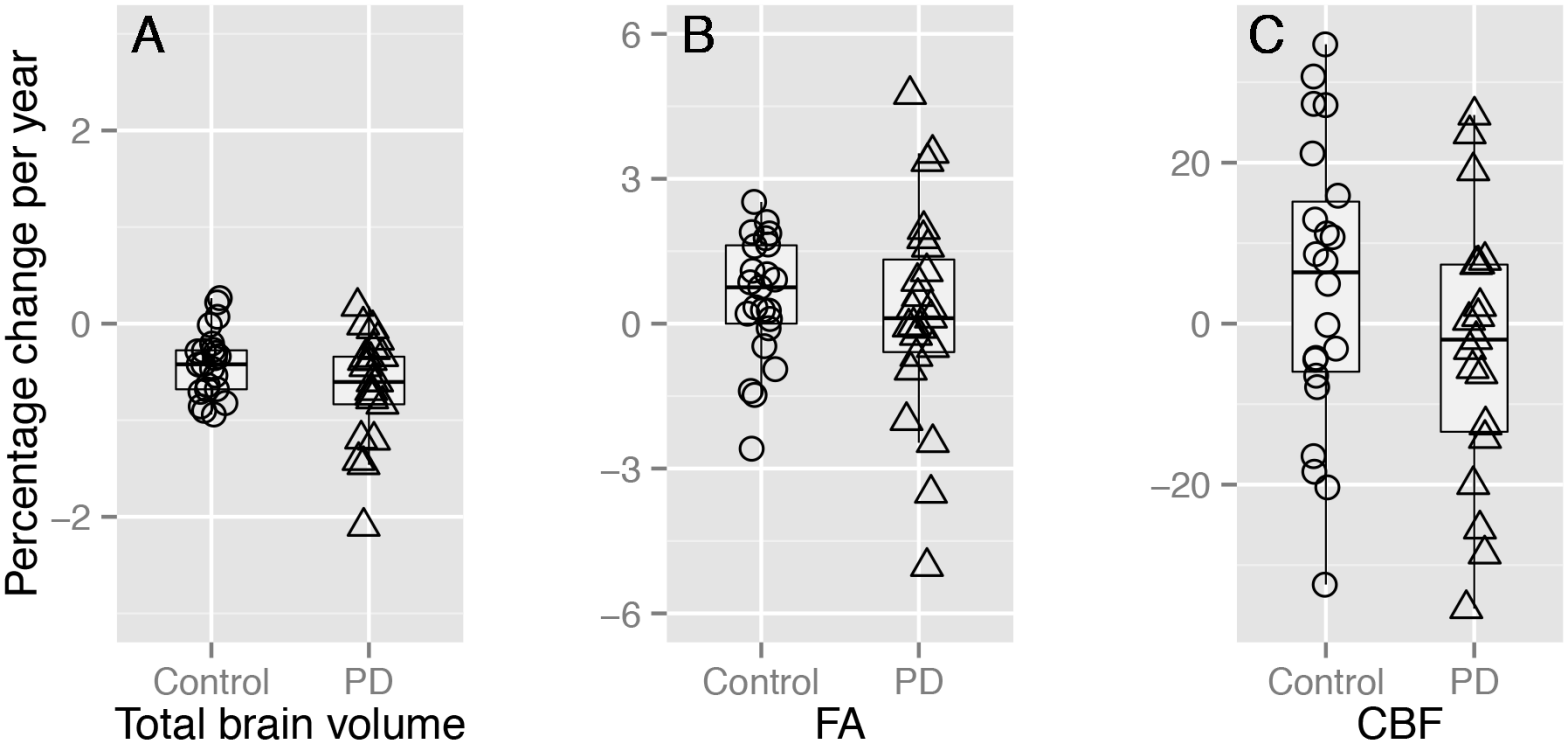
Percentage change in brain metrics over one year. (A) Total brain volume change (%) per year via SIENA, (B) percent FA change per year, and (C) grey matter CBF change per year (%). (A) shows a significant main effect of time in each group whereas (B) and (C) show no significant change; there were no significant group-by-time interactions in upon total brain volume, FA, or CBF change (see Table 3). Note, the y-axis scale increases from [–3 3] in (A), to [–6 6] in (B), to [–35 35] in (C).

Both PD (mean [95% CI], -0.71% [-0.93 –-0.49]) and control groups (-0.48% [-0.70 –-0.25]) showed significant loss of brain tissue over one year (Fig 1A), but this was not significantly different between the groups (Table 3). There were no significant effects of time or group-by-time interactions upon FA (Fig 1B), MD, L1, or RD averaged across the white matter skeleton, or average GM perfusion (Fig 1C and Table 3).

### Voxel-wise results

Group effects: There were no significant differences in GM volume or DTI metrics between groups, but there was lower cerebral perfusion in localized medial posterior parietal areas and right middle temporal gyrus in PD relative to controls (data not shown).

**Fig 2:**
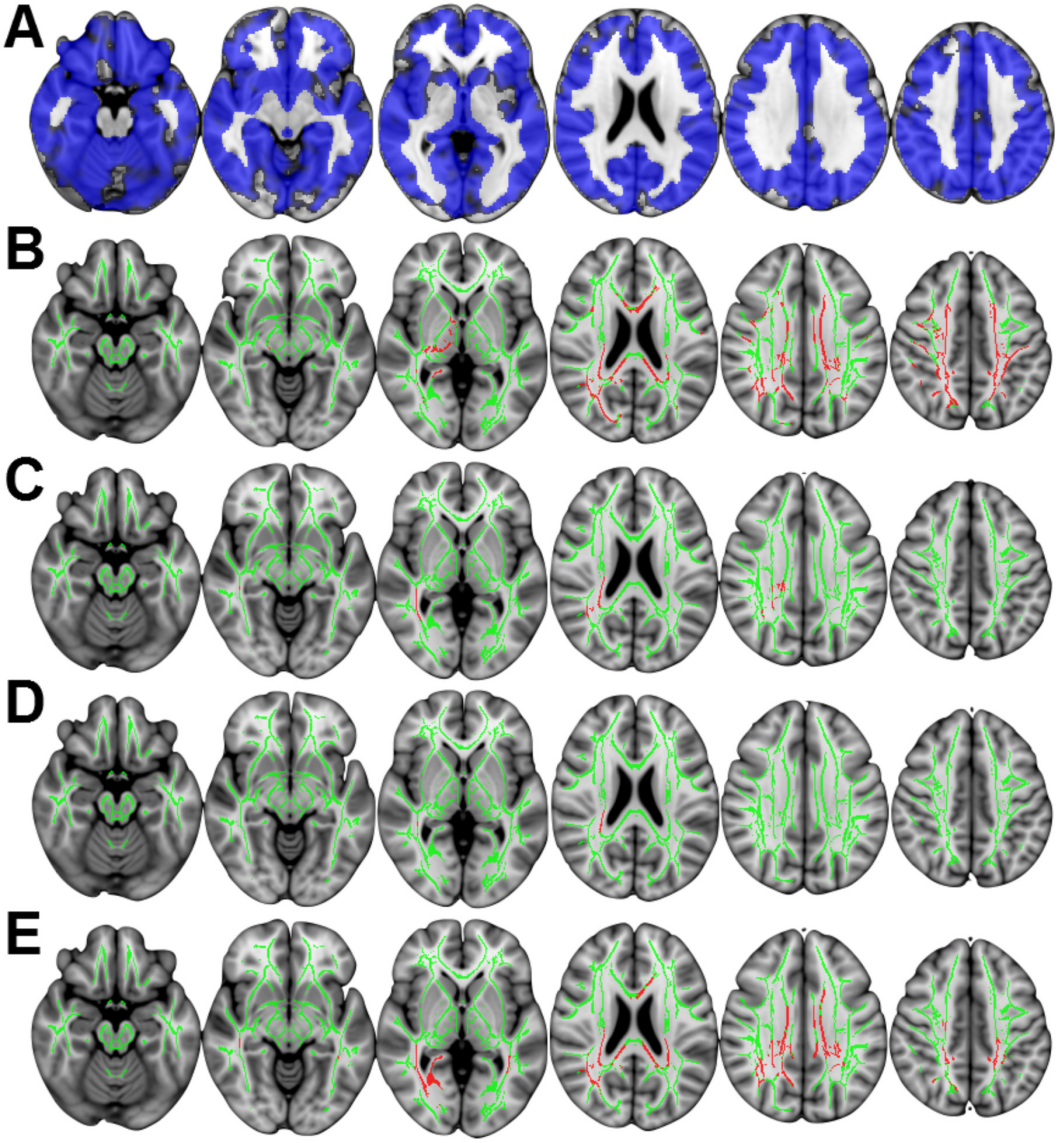
Significant MRI change over time across both groups. (A) Blue indicates significant grey matter atrophy over one year. (B-E) depict results derived from TBSS; the FA skeleton is shown in green, while red indicates significant change over time. (B) Significant reduction in FA over time in multiple white matter tracts. Significant increases in MD (C), L1 (D), and RD (E) over time. MD and L1 showed localized increases in right superior corona radiata. The pattern of increases in RD over time mirrored the regions identified in the FA comparison. All comparisons p<0.05 corrected for multiple comparisons using threshold free cluster enhancement, displayed in radiological convention (right of image is left of brain). Slices displayed: z = -18, -8, 0, 22, 32, 42 mm.

Time effects: In terms of changes across time, irrespective of group, we identified widespread GM atrophy occurring over the one-year period (Fig 2A). FA showed widespread reduction in numerous principal white matter tracts, specifically in genu, body, and splenium of corpus callosum, cingulum bundles, superior corona radiata, and the posterior sections of multiple fasciculi (Fig 2B), while MD, L1, and RD exhibited significant, yet more restricted increases (Fig 2C–2E). We identified no significant change in cerebral perfusion.

**Fig 3:**
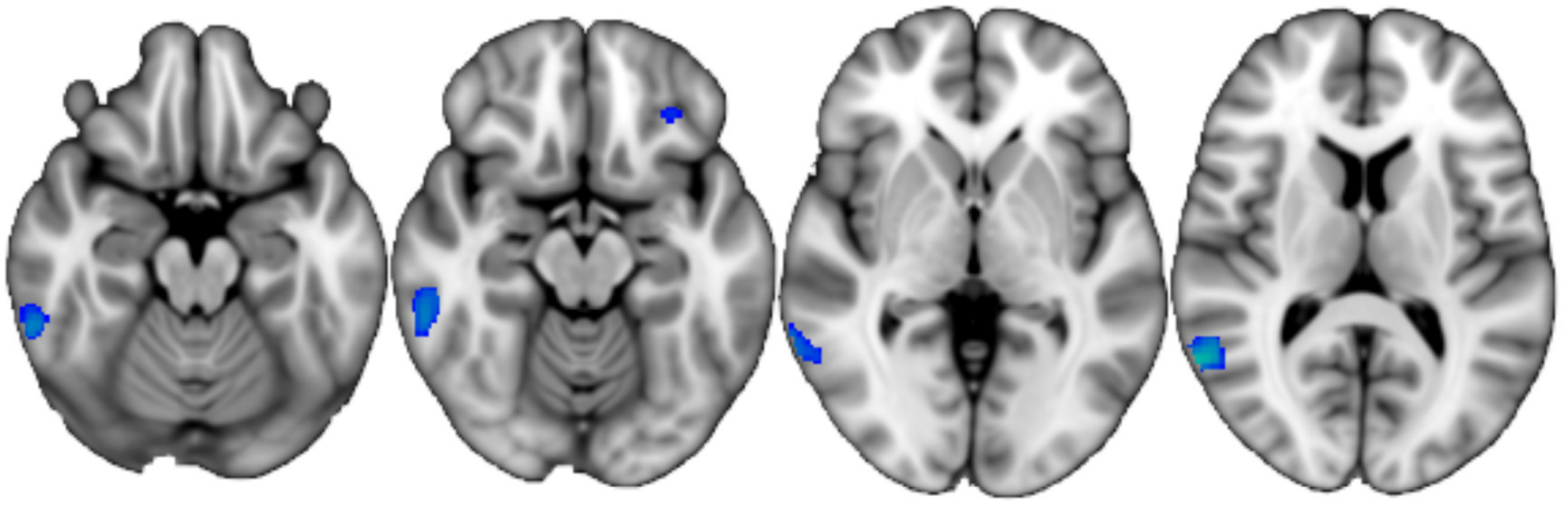
Accelerated atrophy in PD. Blue-green indicates areas where PD showed a higher rate of GM atrophy than controls over one year, overlaid on the MNI152_T1_brain image (slices displayed: z = -18, -14, 0, 12 mm; TFCE-corrected p<0.05; radiological convention).

Group-by-time interactions: The PD group exhibited significantly increased GM atrophy rate (faster loss of tissue) relative to controls in bilateral inferior and right middle temporal gyrus (temporo-occipital region) and left orbito-frontal cortex (Fig 3). When performing voxelwise analysis of DTI metrics along principal white matter tracts using TBSS, there were no significant differences between controls and our sample of PD participants in the rate of change of DTI metrics; there were no voxelwise differences in the rate of perfusion change.

## Discussion

This longitudinal multimodal MRI study investigated progressive changes in a group of older Parkinson’s disease patients with moderate disease burden over one year using structural, diffusion, and perfusion MRI. A voxelwise analysis of structural MRI, but not DTI (via TBSS) or perfusion MRI, revealed significantly greater change in PD relative to matched healthy controls over time. The areas of significantly grey matter atrophy in PD were localized to bilateral inferior and right middle temporal cortex, and left orbito-frontal cortex, consistent with areas of faster cortical thinning reported in PD-MCI [21]. Furthermore, these areas have been associated with more advanced stages of PD [4, 39–41]. However, reports of cerebral tissue loss over time in non-demented PD range from no statistical difference from normal aging [14, 15, 18, 24], to both global and regionally accelerated atrophy in PD [12, 21, 22]. These varying results likely reflect the use of different methodologies, advancement of imaging and processing techniques, length of follow up, and heterogeneity of age and disease severity within PD.

Longitudinal DTI changes in the context of PD are virtually unexplored. Similar to the current study, Rossi et al. [42] failed to identify significant DTI change over two years in PD using TBSS. However, Ofori et al. [24] recently identified longitudinal changes in free-water within the substantia nigra in PD over one year, and these changes predicted longitudinal change in both motor and cognitive outcomes. The differing results may be explained through methodology as well as the composition of the PD groups studied. The current study and Rossi et al. acquired a low number of diffusion encoding directions (28 and 20, respectively) and employed standard DTI processing to investigate voxelwise differences along the centers of principal white matter tracts using TBSS. Ofori et al., [24] on the other hand, acquired a larger number (64) of diffusion directions and employed a more advanced bi-tensor diffusion model, which allows modeling of free water as well as water molecules close to tissue membranes, resulting in FA and MD of tissue compartments, as well as a free-water compartment. Perhaps more relevant, Ofori et al. [24] utilized a region of interest approach focused on an area known to be involved in PD, the substantia nigra, while the current study and Rossi et al.[42] used a whole-brain, hypothesis-free, voxelwise approach. In addition, our sample of PD participants were older and of moderate disease severity, whereas those investigated by Ofori et al. were younger with shorter disease duration. When investigated using traditional DTI models and TBSS in the current study, older PD patients and matched controls exhibited similar rates of DTI change over time, suggesting that any PD-specific changes over one year on this measure does not extend beyond the normal age-associated change. However, future work may benefit by adopting the approach used by Ofori and colleagues; it will be particularly pertinent to determine whether the metrics that arise from the bi-tensor diffusion model are able to detect change in other white matter areas affected by PD progression.

We are unaware of any longitudinal perfusion MRI studies that have tracked PD progression, but radiotracer-based studies suggest that reduced metabolism and perfusion may occur early in the disease process [43]. Perfusion reductions have been identified in non-demented PD relative to controls [44], while converters to PDD exhibited widespread reductions in cortical metabolic activity [45] and perfusion [46] over two years. Huang et al. [47] identified the differential progression of metabolic (FDG) abnormalities associated with motor and cognitive aspects in PD. Imaging PD participants at baseline, then two, and four years later, they showed an increasing expression of a topographic pattern of metabolic activity associated with the motor aspects of PD significantly increased relative to healthy controls at all three timepoints. Conversely, an established PD cognitive pattern only diverged from controls at four years after baseline. Our PD group, although showing regional reductions in perfusion at baseline, underwent no significant perfusion deterioration over a year and showed no significant difference in rate of change between PD and controls groups.

Pseudo-continuous ASL has both adequate test-retest [48] and inter-scanner reliability [49]. However, of the three MR modalities used in our study, ASL had the highest variability. Therefore, our failure to detect perfusion change over time may reflect (a) a small change masked by the short follow-up period or relatively high inter- and intra-subject variability, (b) no underlying perfusion change, or (c) the susceptibility of perfusion MRI to be influenced by short-term, state-dependent changes that may mask underlying long term effects, e.g. the effects of eyes open/closed or drowsiness [50]. While all participants closed their eyes in the present study, we did not monitor drowsiness or other physiological states while in the scanner. It is therefore possible that perfusion changes associated with psychological state masked disease-specific changes of smaller magnitude that may have occurred over one year. This last point may also explain the counterintuitive finding of structural changes in the absence of more ‘functional’ perfusion or microstructural diffusion changes. While one might expect to identify functional and microstructural changes prior to the occurrence of gross atrophy, the perfusion and diffusion measures may be more variable, depending on, for example, physiological state or scanner stability (e.g. field homogeneity). While evidence suggests that early perfusion and metabolic reductions may lead to subsequent atrophy in PD, atrophy and hypometabolism do not coincide in every single cerebral region; for example in some studies the temporal lobe has shown more atrophy than hypometabolism [51]. This may reflect a compensatory mechanism capable of maintaining neuronal activity despite structural damage [51, 52].

Over one year, our PD group remained clinically stable. We measured no significant change in overall motor severity or cognitive impairment in the group as a whole. It is perhaps unsurprising then that the PD group exhibited only localized accelerated brain change. While serial imaging over one year in Huntington’s disease [10], Alzheimer’s disease [53], and multiple sclerosis [11] has revealed increased atrophy relative to controls, one year is a brief epoch relative to the overall duration of PD. Longer follow up periods may be needed to capture divergent trajectories of progressive brain changes. Furthermore, brain deterioration may accelerate in tandem with cognitive impairments, and these impairments generally require greater than one year to manifest in a sample that begins with relatively normal cognition. While cognitive subgroups could not be meaningfully explored in our study (four PD-MCI participants and only one conversion to dementia), recent work has shown areas where cortical thinning occurs faster in PD-MCI [21] and those developing dementia [19].

While we corrected all voxel-wise imaging tests for multiple comparisons across space, we did not explicitly correct for the number of imaging measures utilized. Consistency with recent findings lends support to our results [19, 21], but all group × time interactions disappeared at the more stringent statistical threshold of TFCE-corrected p<0.01. While older and at a moderate stage of disease, most PD patients in this sample were cognitively unimpaired at baseline and only one converted to dementia over the duration of the study (this individual was excluded from the perfusion analysis due to motion). It is possible that PD patients developing dementia may exhibit accelerated brain changes, but this could not be tested in the current study.

All scans were acquired with the same hardware and software versions, bar two baseline scans (both stable PDN) that were acquired before a software upgrade. We did not account for this change in software version as it applied to only two individuals. We do not believe that this small inconsistency would alter conclusions; however this could not meaningfully be tested. Motion influences are always of concern in MRI investigations. We found no difference in motion between PD and controls and no difference in the extent of motion between DTI scanning sessions. Even so, relative motion was included as a covariate in all DTI comparisons, as motion can particularly affect DTI measures [54]. While standard DTI processing steps were followed, the recent work by Ofori et al.[24] suggests that novel, more robust model fitting and analysis pipelines should be adopted for further diffusion work.

In conclusion, both older, PD participants with moderate disease and matched controls showed grey matter atrophy and change in DTI metrics, but not perfusion MRI, over one year. The PD group showed accelerated grey matter loss in bilateral inferior and right middle temporal, and left orbito-frontal cortices relative to controls. However, we identified a similar rate of voxelwise decline across DTI (via TBSS) and no perfusion change. Future studies may benefit from focusing on those at increased risk of developing dementia (e.g. PD-MCI), employing novel diffusion acquisitions and models, and following these patients over longer time periods in order to isolate PD-specific brain patterns of disease and cognitive progression.

## Supporting Information

S1 Data The data and analysis script used. (ZIP)

S1 Table The five cognitive domains and individual tests. (DOCX)
