# Supplementary material for: Tracking Parkinson’s Disease over One Year with Multimodal Magnetic Resonance Imaging in a Group of Older Patients with Moderate Disease
Source: PLoS One. 2015 Dec 29;10(12):e0143923. doi: 10.1371/journal.pone.0143923 (PMC4694717; doi:10.1371/journal.pone.0143923)
Supplement: S1 Data — (ZIP) [file pone.0143923.s001.zip › ReadMe.doc]

Read me file for Supporting Information.

Melzer et al. “Tracking Parkinson’s Disease Over One Year with Multimodal Magnetic Resonance Imaging.”

File 1: Melzer_FU1year_PLOS_Rscript.txt. This is a text file of an R script used to analyze the data in File 2 (Melzer_FU1year_PLOS.csv). The script contains steps to re-create the analysis, table values, and Fig1.

File 2: Melzer_FU1year_PLOS.csv. This spreadsheet holds the data used in the manuscript. The following list explains column names:

subject: Each subject has a unique letter identifier

AnonID: Anonymous ID

Group: PD or Control

Category: Cognitive category. PDN = PD with normal cognition, PD-MCI = PD with Mild Cognitive Impairment, PDD = PD with dementia, HC = healthy control.

Category_HC-MCI: Cognitive category. However, this time 3 controls classified as MCI are identified (C-MCI).

Convert: The status of each individual at 1 year follow up. HC = healthy control, Stable = Did not develop dementia, Convert = Developed dementia at 1 year.

Age_at_scan_years: Age at assessment/scan

Age_base: Age as study entry

time_btw_scans_years: Time between scans measured in years.

Female: 0 = male, 1 = female

Education: years of education

FU_1year: 1^st^ or 2^nd^ scan

time: assessment time, either baseline (study entry) or follow up (~ 1year).

time.y: time, in years, of follow up.

SIENA: % brain change between scans. Values only at baseline (first timepoint).

DTI relative motion: average relative motion between adjacent diffusion volumes (see manuscript for details).

mean FA/MD/L1/RD: mean FA /MD/L1/RD values along the white matter skeleton

CBF_gm: mean grey matter perfusion (ml/100g/min), including cortex and subcortical grey.

diff.FA.Y0-Y1 (same for MD/CBF): raw difference between mean FA at Y0 and Y1.

per.diff.FA.Y0-Y1 (same for MD/CBF): percent difference between mean FA at Y0 and Y1.

per.diff.year.FA.Y0-Y1 (same for MD/CBF): percent difference between mean FA at Y0 and Y1, divided by the time between Y0 and Y1.

MoCa: Montreal cognitive assessment—global cognitive screen.

UPDRS Part 3: Unified Parkinson’s disease rating scale, part 3, the motor score (only scores for PD participants).

LED: Levodopa equivalent dose.

Attention Total: average z score for tests in the domain of attention, working memory, and processing speed.

Executive Function Total: average z score for tests in the domain of executive function.

Visuo-Total: average z score for tests in the domain of visuospatial/visuoperceptual function.

Language all Domains: average z score for tests in the domain of Language.

Total all Domains: global cognitive score: average z score for the domain scores (attention, executive function, learning & memory, and visuospatial/perceptual)
