# Supplementary material for: Tracking Parkinson’s Disease over One Year with Multimodal Magnetic Resonance Imaging in a Group of Older Patients with Moderate Disease
Source: PLoS One. 2015 Dec 29;10(12):e0143923. doi: 10.1371/journal.pone.0143923 (PMC4694717; doi:10.1371/journal.pone.0143923)
Supplement: S1 Table — (DOCX) [file pone.0143923.s002.docx]

| **Supplementary Table 1: The five cognitive domains and individual tests** | | | | |
| --- | --- | --- | --- | --- |
| **Attention, working memory and processing speed** | **Executive function** | **Visuoperceptual/**  **visuospatial** | **Learning and memory** | **Language** |
| Digits forward/backward | Action (verb) fluency | Judgment of line orientation | CVLT-II SF acquisition ^a^ | Boston naming test |
| Digit ordering | Letter fluency  (D-KEFS) | Fragmented letters | CVLT-II SF short delay (30secs)^b^ | DRS-2 similarities sub-tests |
| Map test (test of everyday attention) | Category fluency  (D-KEFS) | Rey complex figure copy | CVLT-II SF long delay (10mins)^b^ | ADAS-Cog (object and finger naming, commands, comprehension, spoken language and word finding difficulties) |
| Stroop color reading | Category switching  (D-KEFS) | Picture completion | Rey – short delay (3mins)^c^ |  |
| Stroop word reading | Trails B |  | Rey – long delay (30mins)^c^ |  |
| Trails A | Stroop interference  (D-KEFS) |  |  |  |
| ADAS-Cog, Alzheimer’s Dementia Assessment Scale; CVLT-II SF, California Verbal Language Test-II Short Form; D-KEFS, Delis-Kaplan executive function system; DRS-2; Dementia Rating Scale-2. | | | | |
